# A New Cytotoxic 19-Nor-cardenolide from the Latex of *Antiaris toxicaria*

**DOI:** 10.3390/molecules14093694

**Published:** 2009-09-21

**Authors:** Hao-Fu Dai, Yu-Juan Gan, Dong-Mei Que, Jiao Wu, Zhen-Chang Wen, Wen-Li Mei

**Affiliations:** 1 Key Laboratory of Tropical Crop Biotechnology, Ministry of Agriculture, Institute of Tropical Bioscience and Biotechnology, Chinese Academy of Tropical Agricultural Sciences, Haikou 571101, China; E-Mails: hfdai@yahoo.cn (H.-F.D.); ganyujuan2006@yahoo.com.cn (Y.-J.G.); snowque@163.com (D.-M.Q.); wujiao2008@yahoo.com.cn (J.W.); 2 Key Laboratory of Tropic Biological Resources of Ministry of Education, Hainan University, Haikou 570228, China; E-Mail: wenzc789@126.com (Z.-C.W.)

**Keywords:** *Antiaris toxicaria*, toxicarioside H, nor-cardenolide, cytotoxicity

## Abstract

A new nor-cardenolide, named toxicarioside H (**1**), was isolated from the latex of *Antiaris toxicaria* (Pers.) Lesch (Moraceae). Its structure was elucidated on the basis of HRFAB-MS and spectroscopic techniques (IR, UV, 1D and 2D NMR). Compound **1** showed significant cytotoxicity against K562, SGC-7901, SMMC-7721, and HeLa cell lines *in vitro* by MTT method.

## Introduction

The latex of *Antiaris toxicaria* (Pers.) Lesch (Moraceae) has been the main source of the poisonous principle in dart and arrow poisons prepared throughout southeastern Asia, from Burma to China and Indonesia [1]. Its toxicity is due to the presence of a complex mixture of cardenolide glycosides [2,3]. Previous studies of the toxicity of this plant in Indonesia or Malaysia led to the isolation of cardenolides from the latex, seeds, and stems [4,5]. In our previous work on screening for cytotoxic agents from tropical medicinal plants in Hainan Province, China, three new cytotoxic cardenolides named toxicarioside E, F, and G have been isolated from the latex of *A. toxicaria* [6,7]. In continuation of our search for cytotoxic constituents, a new nor-cardenolide, named toxicarioside H (**1**) was obtained. Compound **1** showed potent growth-inhibitory activity on K562, SGC-7901, SMMC-7721, and HeLa cell lines. The present paper discusses the structural elucidation and cytotoxicity of this new compound.

## Results and Discussion

Compound **1** was obtained as a white amorphous powder. The ion peak [M－H]^−^ at *m/z* 567.2846 in the high-resolution FAB-Mass spectrum corresponded to the molecular formula C_29_H_44_O_11_ (calcd. for [M－H]^−^ 567.2889). This formula could also be validated through the ^1^H-NMR, ^13^C-NMR (DEPT) spectra. The IR spectrum displayed absorptions for hydroxyl (3,444 cm^−1^), conjugated carbonyl (1,740 cm^−1^), and double bond (1,627 cm^−1^) moieties. In the ^1^H-NMR spectrum, the signal at *δ* 5.90 (s, 1H, H22) and signals at *δ* 4.98, 4.89 (each 1H, *J*_AB_ = 18.6 Hz, H21a, H21b) suggested the presence of the characteristic butenolide of a cardenolide system. Other prominent signals included a high field methyl singlet at *δ* 0.80 (H-18), also suggestive of a cardenolide nucleus, an anomeric proton signal at *δ* 4.75 (d, 1H, *J* = 7.9 Hz, H1') in the ^1^H-NMR spectrum indicated that **1** was a glycoside incorporating a sugar unit with *β*-linkage. Comparing the 1D-NMR spectral data with those of the toxicarioside A showed that the aglycone of **1** had no aldehyde group [8], while C-10 (*δ* 75.2) was substituted by a hydroxyl group. This was further confirmed by the correlations from H2 (1.84, 1.64) and H4 (2.02, 1.38) to C10 (*δ* 75.2) in the HMBC spectrum. Thus, the aglycone of **1** was determined as 10-hydroxy-19-nor-antiarigenin.

The sugar unit of **1** was identified mainly by analysis of ^1^H-NMR and ^1^H-^1^H COSY spectra. ^1^H-^1^H COSY spectrum of **1** allowed unambiguous assignment of the entire H1'- H6' spin system, the relative configuration of C1'-C5' centers could be deduced by analysis vicinal coupling constants for the sugar proton signals. Thus, a H1'/H2' diaxial relationship was indicated by the 7.9 Hz coupling constant observed at H1', while the small, second coupling constant observed in the well resolved double doublet signal for H2' (*δ* 3.01, *J* = 7.9, 2.8 Hz) demonstrated that H3' was equatorial. Likewise, the small coupling constant observed in the triplet signal for H3' (*δ* 4.24, *J* = 2.7 Hz) demonstrated that H4' was axial, and the large coupling constant observed in the double doublet signal for H4' (*δ* 3.14, *J* = 9.6, 2.8 Hz) demonstrated that H5' was also axial. In the HMBC spectrum, the correlation from H-OCH_3_ (*δ* 3.41) to C2' (*δ* 81.8) (Figure 2) suggested that C2' was the site for attachment of the O-methyl ether. Hence, the sugar moiety was determined as a rare sugar javose. The HMBC correlations between H3 (*δ* 4.11) and C1' (*δ* 98.1) (Figure 2) suggested that the sugar moiety was linked to C3. The relative stereochemistry of **1** was determined by ROESY correlations (Figure 2). The sugar moiety of **1** was determined as D-javose by acid hydrolysis. Based on the above evidence, compound **1** was identified as 10 *β*-hydroxy-19-nor-antiarigenin-3-*O*-*β*-D-javopyranoside, named toxicarioside H.

**Figure 1:**
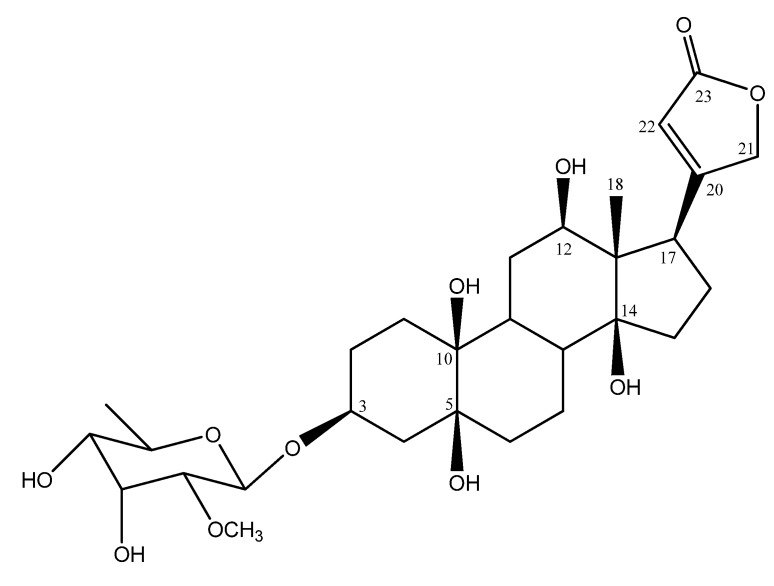
Structure of compound **1**.

**Figure 2:**
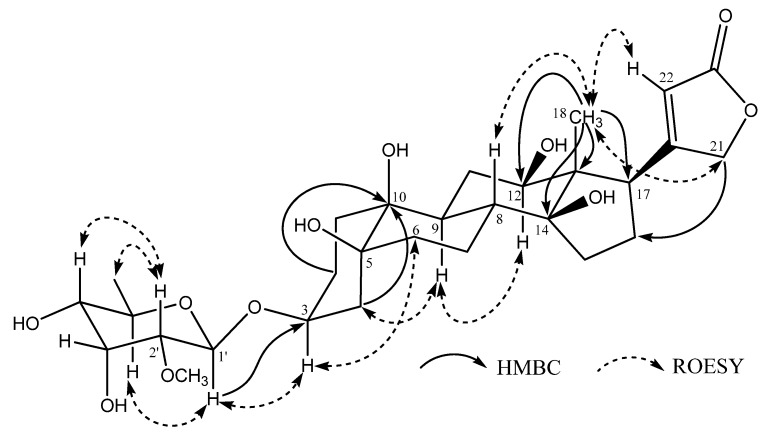
Key HMBC and ROESY interactions for compound **1**.

In addition to the effect of the cardenolides on the activity of the ubiquitous cell surface enzyme Na^+^/K^+^-ATPase, recent studies have demonstrated that this kind of compounds could inhibit the growth of cancer cells. We have, therefore, tested the isolated compounds for their cytotoxicity. The cytotoxicity of compound **1** was evaluated *in vitro* using the MTT method [9]. Compound **1** showed significant cytotoxicity against the K562, SGC-7901, SMMC-7721, and HeLa cell lines with the IC_50_ values of 0.004−0.037 μg/mL, and mitomycin C was used as a positive control.

## Experimental

### General

Melting points were obtained on a Beijing Taike X-5 stage apparatus and are uncorrected. Optical rotation was recorded using a Rudolph Autopol III polarimeter (Rodolph Research Analytical, New Jersey, USA). The UV spectra were measured on a Shimadzu UV-2550 spectrometer. The IR spectra were obtained on a Nicolet 380 FT-IR instrument, as KBr pellets. The NMR spectra were recorded on a Bruker AV-400 spectrometer, using TMS as an internal standard. The HRFABMS spectra were measured with a VG Auto-Spec-3000 mass spectrometer. Column chromatography was performed with silica gel (Marine Chemical Industry Factory, Qingdao, P.R. China). TLC was preformed with silica gel GF254 (Marine Chemical Industry Factory, Qingdao, P.R. China).

### Plant material

Latex of *Antiaris toxicaria* (Pers.) Lesch was collected in Lingshui county of Hainan Province, China in November 2005, and the plant was identified by Professor Zhu-Nian Wang. A voucher specimen (No. AN200511) is deposited in the Institute of Tropical Bioscience and Biotechnology, Chinese Academy of Tropical Agricultural Sciences.

### Extraction and isolation

Four L of latex of *A. toxicaria* was extracted three times with 95% EtOH (eight L) at room temperature and then filtered. The combined extract was evaporated *in vacuo* to yield a syrup (263.8 g), which was suspended in H_2_O and partitioned successively with petroleum ether and EtOAc to afford a petroleum ether extract and EtOAc extract. The EtOAc extract (8.68 g) was chromatographed over a silica gel column by gradient elution utilizing CHCl_3_ and MeOH as solvent system to give sixteen fractions. After repeated silica gel column chromatography (CHCl_3_-MeOH 14:1), fraction 15 (1.24 g) afforded compound **1** (11.3 mg).

### Toxicarioside H *(**1**)*

White amorphous powder. M.p. 205−208 °C; [*α*]^23^_D_ = −1.8 (*c =* 1.0, MeOH); UV (MeOH) λ_nm_ (logε): 214 (4.28); IR (KBr) ν_max_ (cm^−1^): 3,444, 2,925, 2,361, 1,740, 1,625, 1,077; ^1^H- and ^13^C-NMR spectral data see Table 1; HRFABMS (neg.) *m/z*: 567.2846 [M－H]^−^ (calcd. for C_29_H_44_O_11_, 567.2889).

**Table 1:** NMR data of **1** in CD_3_OD (^1^H: 400 MHz; ^13^C: 100 MHz; *δ* in ppm, *J* in Hz).

Position	δ_H_	δ_C_	Position	δ_H_	δ_C_
1	1.87, 1.11 (each 1H, *m*)	24.7	16	2.13, 1.92 (each 1H, *m*)	28.4
2	1.84, 1.64 (each 1H, *m*)	29.2	17	3.34 (1H, br s)	46.9
3	4.11 (1H, br s)	74.9	18	0.80 (3H, s)	9.8
4	2.02, 1.38 (each 1H, *m*)	35.8	20		178.6
5		75.4	21	4.98, 4.89 (each 1H, d, 18.6 Hz)	75.4
6	2.03, 1.84 (each 1H, *m*)	35.7	22	5. 90 (1H, s)	117.8
7	1.92, 1.67 (each 1H, *m*)	26.9	23		177.4
8	1.81 (1H, m)	40.6	1'	4.75 (1H, d, 7.9 Hz)	98.1
9	1.59 (1H, m)	37.9	2'	3.01 (1H, dd, 7.9, 2.8 Hz)	81.8
10		75.2	3'	4.24 (1H, t, 2.7 Hz)	68.7
11	1.69, 1.60 (each 1H, *m*)	30.5	4'	3.14 (1H, dd, 9.6, 2.8 Hz)	74.2
12	3.37 (1H, dd, 10.6, 4.1 Hz)	75.6	5'	3.72 (1H, m)	70.8
13		56.9	6'	1.23 (3H, d, 6.2 Hz)	18.6
14		86.4	2'-OCH_3_	3.41 (3H, s)	57.0
15	1.92, 1.70 (each 1H, *m*)	33.3			

### Acid hydrolysis of ***1***

Compound **1** (4.0 mg) was dissolved in MeOH (2.0 mL) and 5% H_2_SO_4_ soln. (2.0 mL) and hydrolyzed under reflux for 3 h. The mixture was allowed to cool, diluted twofold with distilled water, and partitioned between EtOAc and H_2_O. The aq. layer was neutralized with aq. NaHCO_3_ soln. (1.0 M) and evaporated to give sugar residue. The optical rotation of sugar was measured as [*α*]^23^_D_ = − 43 (*c* = 0.12, H_2_O).

### Cell cultures and in vitro cytotoxicity assay

The human myeloid leukemia cell line (K562), human gastric cell line (SGC-7901), human hepatoma (SMMC-7721), and human cervical cancer (HeLa) cells were obtained from the Cell Bank of Type Culture Collection of Chinese Academy of Sciences, Shanghai Institute of Cell Biology. Cells were maintained in RPMI-1640 supplemented with 10% fetal bovine serum (FBS), 100 units/mL penicillin and 100 μg/mL streptomycin sulfate at 37 ºC, 5% CO_2_. The MTT assay was performed according to the method described in previous literature [9]. The IC_50_ values are listed in Table 2.

**Table 2:** *In vitro* cytotoxicities of compound **1** (IC_50_ values, *µ*g·mL^-1^).

Compound	K562	SGC-7901	SMMC-7721	HeLa
**1**	0.037	0.012	0.004	0.007
Mitomycin C *^a^*	7.1	8.8	2.2	6.3

*^a^* Positive control.
